# Supplementary figures and images for: PARP Inhibitor Inhibits the Vasculogenic Mimicry through a NF-κB-PTX3 Axis Signaling in Breast Cancer Cells
Source: Int J Mol Sci. 2022 Dec 18;23(24):16171. doi: 10.3390/ijms232416171 (PMC9785325; doi:10.3390/ijms232416171)

7-AAD

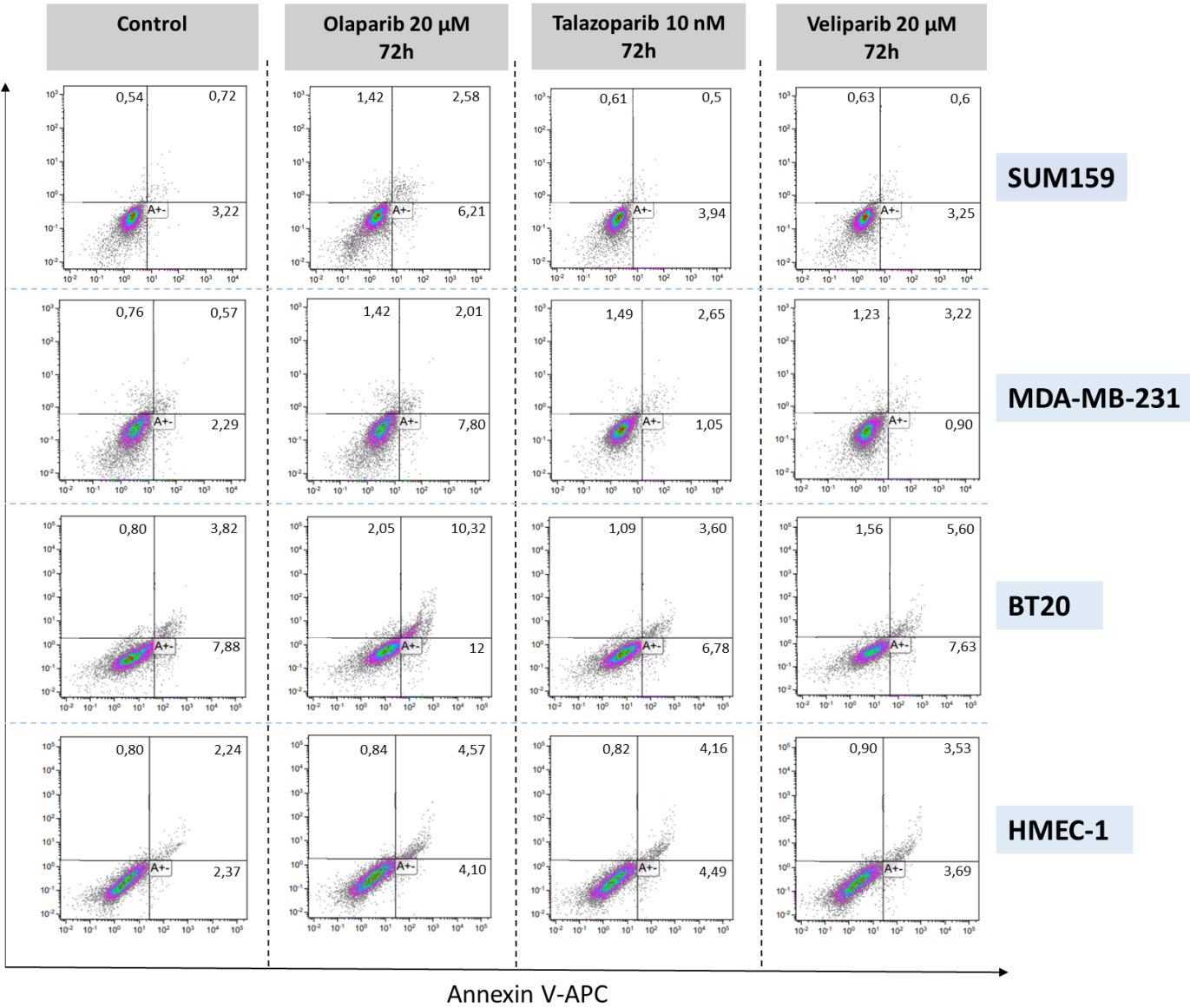

Supplement: Supplementary file 1 [file ijms-23-16171-s001.zip › Supplemental Files/Supplemental S1.pdf]
